# Regulation of Brassinosteroid Signaling and Salt Resistance by SERK2 and Potential Utilization for Crop Improvement in Rice

**DOI:** 10.3389/fpls.2020.621859

**Published:** 2020-12-10

**Authors:** Nana Dong, Wenchao Yin, Dapu Liu, Xiaoxing Zhang, Zhikun Yu, Wei Huang, Jihong Liu, Yanzhao Yang, Wenjing Meng, Mei Niu, Hongning Tong

**Affiliations:** National Key Facility for Crop Gene Resources and Genetic Improvement, Institute of Crop Sciences, Chinese Academy of Agricultural Sciences, Beijing, China

**Keywords:** brassinosteroid, salt stress, rice, gain size, SERK2

## Abstract

The complex roles of the steroid hormone brassinosteroids (BRs) in many different yield- and stress-related traits make it difficult to utilize the hormones for crop improvement. Here, we show that SERK2 as a BR signaling component is a potentially useful candidate for BR manipulation in rice. We generated multiple mutant alleles of *SERK2* by CRISPR/Cas9 editing and show that knockout of *SERK2* results in a compact structure accompanied with increased grain size. SERK2 is localized on plasma membrane and can interact with OsBRI1, the BR receptor, suggesting its conserved role as co-receptor in BR signaling. Consistently, the mutant has impaired BR sensitivity compared to wild type. Notably, the mutant is highly sensitive to salt stress as evaluated by plant survival rate as well as transcriptome analysis, whereas has slightly increased sensitivity to ABA, the stress hormone. By contrast, overexpression of *SERK2* significantly enhances grain size and salt stress resistance, importantly, without affecting plant architecture. Furthermore, while salt suppresses *SERK2* transcription, the protein is greatly induced by salt stress. Taken together, we propose that the adverse condition induces SERK2 accumulation to enhance early BR signaling on plasma membrane in favor of the anti-stress response. Our results illustrate the great potentials of specific BR components such as SERK2 for crop improvement by utilizing flexible strategies.

## Introduction

Brassinosteroids (BRs) are a class of steroid hormones having chemical structures similar to those in animals or insects. Brassinolide (BL), one of the most active BRs, was identified forty years ago, quite later than the discovery of five other classical phytohormones including auxin, cytokinin, gibberellin, abscisic acid (ABA), and ethylene (Grove et al., 1979). BRs were thus considered as the sixth class of phytohormones. Although with a short history, our understanding of BR synthesis and signaling is rapidly progressed. So far, the BR signaling pathway could be one of the best characterized hormone signaling pathways in the model plant Arabidopsis (*Arabidopsis thaliana*). In the prevalent BR signaling model, BRs are perceived on plasma membrane by BRI1-BAK1 receptor complex and the signal is then transduced sequentially by BSK1 kinase, BSU1 phosphatase and BIN2 kinase, eventually leading to activation of BZR1/BES1 transcriptional factors (Kim and Wang, 2010). Each of these components represents a family of protein members presuming to have at least partially redundant functions.

In recent years, many additional players, including a number of interacting proteins with the above-mentioned components, have been identified to be involved in BR signaling (Nolan et al., 2020). Interestingly, it has been shown that PP2a, a phosphatase, simultaneously targets BRI1 and BES1 to regulate BR signaling (Tang et al., 2011; Wu et al., 2011). In addition, some of the BR signaling components, such as BSU1-family members and BZR1-family proteins, could have other functions not associated with BR signaling (Maselli et al., 2014; Chen et al., 2019). Intriguingly, the downstream BIN2 can conversely phosphorylate and regulate BSK-proteins (Sreeramulu et al., 2013; Ren et al., 2019). Since BSK-proteins can interact with a number of BR-signaling components, the scaffold function of the proteins has been proposed (Ren et al., 2019). These studies largely advanced our understanding of the BR signaling, and suggested a complicated signal tuning system underlying the important hormones in plants.

In rice, BRs play predominant roles in regulating many important agronomic traits, including plant height, grain size, and leaf erectness. Thus, BRs are considered to have great potentials in crop improvement (Tong and Chu, 2018). Interestingly, a few studies have shown that rice could have a different BR signaling pathway, at least at some steps, from that in Arabidopsis. For example, PPKL1 has been shown to negatively regulate BR signaling, in contrast to its ortholog BSU1 in Arabidopsis (Gao et al., 2019). And also, BRs could have different functions in different species. For example, BRs significantly regulate photo-morphogenesis in Arabidopsis but appear to play a minor role in pea (Bishop, 2003). Therefore, to effectively utilize BRs in crops, the hormone functions should be carefully evaluated in each species.

Given that rice is grown in a largely different environment (i.e., temperature, water, light) compared with Arabidopsis, whether BRs differentially function in regulating stress responses in various species should be particularly concerned. However, despite the rapid progress made in BR-regulated plant growth and development, our understanding of BR-regulated stress response remains very limited, especially in rice. For example, several studies in Arabidopsis have respectively shown that BRs favor the plant tolerances to salt and cold (Sharma et al., 2013; Ye et al., 2019) but suppress drought tolerance (Chen et al., 2017). In addition, a number of studies in Arabidopsis also revealed that there exists multilayer crosstalk between BRs and ABA, the well-known stress hormone. For example, the ABA signaling phosphatase can dephosphorylate BIN2 to regulate plant growth and development (Wang et al., 2018), whereas BIN2 can phosphorylate both SnRK2 and ABI5, the ABA signaling components, to modulate stress responses and seed germination (Cai et al., 2014; Hu and Yu, 2014). However, little is known about how BRs regulates stress responses in rice.

BAK1, as the BR co-receptor, belongs to SERK-family proteins. Beside BR signaling, SERK-members play diverse roles in various biological processes such as embryo development, senescence, and plant immunity (Fan et al., 2016). In rice, OsBAK1 has also been suggested to play a conserve role as its ortholog in Arabidopsis, because knockdown or knockout of *OsBAK1* resulted in a compact plant structure and small grains (Li et al., 2009; Yuan et al., 2017). A couple of other proteins, such as a G protein and a remorin protein, have been shown to interfere with OsBRI1-OsBAK1 interaction to regulate BR signaling (Gui et al., 2016; Zhang et al., 2016). In addition, the role of SERKs in plant immunity has also been studied in rice (Chen et al., 2014; Zuo et al., 2014). However, solid genetic evidence supporting the roles of each SERK member in BR signaling or plant growth and development remains scarce. Despite of the intense crosstalk between BIN2 and ABA signaling components, whether and how SERKs, as potentially early BR signaling components, regulate stress responses remains largely unclear. Addressing this question has particular significance for utilizing BRs to simultaneously improve crop yield and stress resistance.

We previously proposed that molecular design utilizing functional specificities of BR-related components is one of potential strategies for BR application (Tong and Chu, 2018). In the scenery, manipulation of a specific BR component could obtain desired traits without negative effects or with even beneficial effects on other traits. In an attempt to screen this kind of genes, we have generated a panel of mutant defective of putative BR signaling genes by CRISPR/Cas9 editing. In this study, we evaluated the roles of *SERK2* and showed that *SERK2* could be one of the candidates meeting our requirement. The *serk2* mutants had reduced plant height and compact structure, whereas accompanied with increased grain size, thus the gene could be useful for engineering plant architecture without negative effect on grain size. On the other hand, we showed that overexpression of *SERK2* can simultaneously enhance salt resistance and grain size, without negative effect on plant architecture. Thus, the gene could be valuable for crop improvement in rice.

## Materials and Methods

### Plant Materials and Growth Condition

A *japonica* rice (*Oryza sativa*) cultivar, Zhonghua11 (ZH11), was used as background for all the analyses. Plants were grown either in field under natural conditions or in a growth chamber (GXZ-800D) with settings: 30°C day and 28°C night, 10-h/14-h light/dark cycle. When grown in the chamber, half-strength Murashige and Skoog basal salt mixture (1/2MS) was used as nutrient source. Grain size is measured using an integrated photographing and analysis system (WSeen SC-G).

### Gene Expression Analysis

Total RNA was isolated from the samples collected using Trizol (Invitrogen), and cDNA was prepared using a reverse transcription kit (Toyobo) following the product instructions. Quantitative real-time PCR (qRT-PCR) was performed on a LightCycler 96 system (Roche) using SYBR Green PCR mix (Roche). The gene expression levels were normalized to the transcript level of the reference gene *Ubiquitin*. Primers used for qRT-PCR are listed in Supplementary Table 1

### Vector Construction and Transgene

The *serk2* mutants were produced using CRISPR/Cas9 system (Lu et al., 2017) targeting 5′-TGGGACAATACCTAATGAA CTGG-3′ corresponding to 333-355 of the *SERK2* coding sequence. For overexpression analysis, the full-length cDNA of *SERK2* was amplified and then introduced into an empty vector named *p1300-35S-FLAG* by in-fusion technology (Clontech). See Supplementary Table 1 for primers used for vector construction. The resulted vector *p1300-35S-SERK2-FLAG* was introduced into ZH11 callus to produce the overexpression plants by *Agrobacterium*-mediated method.

### Subcellular Localization Analysis in Protoplast

Rice protoplasts were prepared according to the previous description (Bart et al., 2006). A vector *p2300-35S-SERK2-GFP* expressing SERK2-GFP fusion protein was used to transfecting rice protoplast. The empty vector expressing sole GFP was used as control and a vector expression plasma-membrane localized protein was used as reference. See Supplementary Table 1 for primers used for vector construction. After one-night incubation, the protoplast cells were observed under a confocal fluorescence microscope (Zeiss) and those emitting fluorescence were photographed.

### Split-luciferase Complementation Analysis

Empty vectors for this analysis have been introduced previously (Chen et al., 2008). SERK2 and OsBRI1 were introduced into the empty vectors, resulted in *p1300-split-nLUC* (nLUC-BRI1) and *p1300-split-cLUC* (cLUC-SERK2) respectively, which were used for luciferase complementation analysis to test the potential interactions in tobacco (*N. benthamiana*) leaves. See Supplementary Table 1 for primers used for vector construction. The vectors were transformed into agrobacteria and the desired couples were mixed and then infiltrated into tobacco leaves as detailed previously. Chemiluminiscence was photographed using an imaging system equipped with a cold CCD (NightSHADE LB985).

### Stress and Hormone Treatment

One-week-old seedlings were treated with 200 mM NaCl for 6-8 days and recovered for 3 days to count survival frequency, or treated for different times for transcription and protein analyses. Sterilized seeds were sown on 1/2MS agar medium with or without ABA (Sigma-Aldrich) supplementation and grown for 10 days prior to root and shoot measurements. Different concentrations of BL were used for BR sensitivity test. Briefly, BL was dissolved in ethanol for leaf inclination assay (0, 10, 100, and 1000 ng) and dissolved in DMSO for cultivation on agar medium (0, 0.1, and 1 μM) to test the shoot and root growth as described previously (Tong and Chu, 2017). For molecular analysis, one-week-old seedlings were treated with 1 μM BL for different times.

### RNA Sequencing

Four samples, including ZH11, *serk2*, salt-treated ZH11, and salt-treated mutant (200 mM NaCl, 8 h), were prepared for transcriptome analyses. The aerial parts of one-week-old seedlings were harvested for total RNA extraction. The purified RNAs were used for library construction using a NEBNext Ultra RNA Library Prep Kit for Illumina (NEB). Three biological replicate libraries were prepared for each sample. Clustering of index-coded samples was performed on a cBot Cluster Generation System using a Nova-seq Cluster Kit (Illumina). The libraries were sequenced on the Illumina NovaSeq S6000 platform and 150 bp paired-end reads were generated. FDR (false discovery rate) value < 0.01 and log2FC (fold change) ≥ 1 were used to identify differentially expressed genes (DEGs). Analysis of overlapping DEGs was performed online using Venny^1^. Complete DEG lists used for overlapping analysis were provided in Supplementary Tables 2, 3.

### Immunoblotting

The *SERK2*-overexpression plants were treated with salt or BL for different times, and the seedling shoots were harvested for immunoblotting analysis. Total proteins were extracted as described previously. Commercialized anti-Flag (1:2,000, Sigma-Aldrich) and anti-HSP (1:5,000, BPI) antibodies were used for immunoblotting analyses on a Trans-Blot Turbo Transfer System (Bio-Rad) according to the manufacturer’s instructions.

## Results

### The *serk2* Mutants Show Compact Structure but Increased Grain Size

**FIGURE 1:**
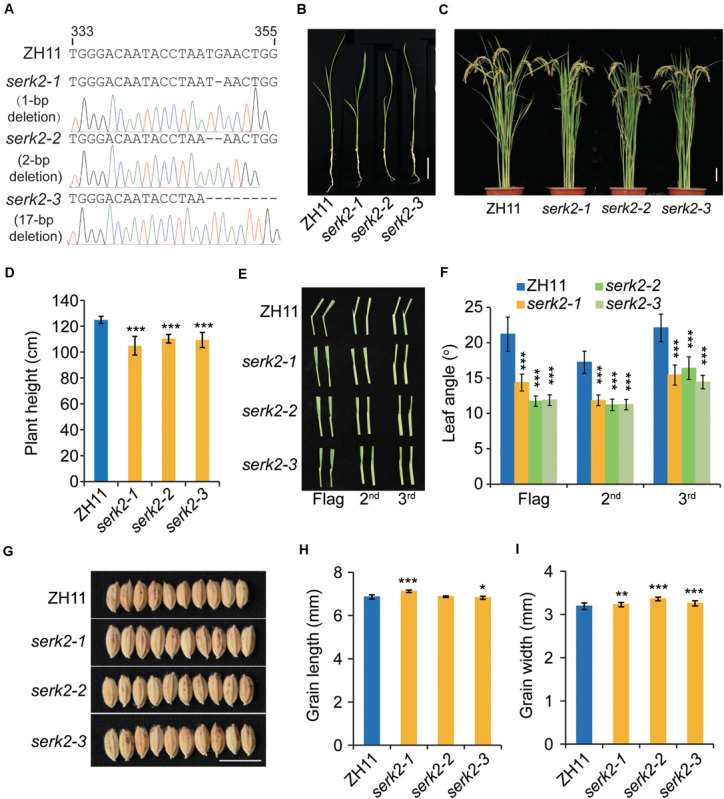
The *serk2* mutants show compact structure accompanied with increased grain size. **(A)** Information of editing site and the mutations. The sgRNA sequence is shown with the position at the CDS indicated. The sequencing chromatograms are also shown. **(B)** Seedling phenotype after 14-d growth. Scale bar = 5 cm. **(C)** Gross morphology of ZH11 and *serk2* mutants. Scale bar = 10 cm. **(D)** Statistical data of the plant height. Bars indicate standard deviation (SD), *n* = 15, ***p < 0.001 by *t*-test. **(E)** Comparison of leaf angles in ZH11 and the mutants. The top three leaves were shown. **(F)** Statistical data of the leaf angles. Bars = SD, *n* = 20, ***p < 0.001 by *t*-test. **(G)** Grain morphology of ZH11 and *serk2* mutants. Scale bar = 1 cm. **(H,I)** Statistical data of the grain length **(H)** and grain width **(I)**. Bars = SD, *n* = 50, ***p < 0.001, **p < 0.01, and *p < 0.05 by *t*-test.

The *serk2* mutants were generated by targeting a specific sequence within *SERK2* (*LOC_Os04g38480*) coding region (Figure 1A). A number of allelic mutants containing various mutations at the targeting site were obtained. Three representative lines containing frame-shift mutations, designated *serk2-1* to *serk2-3*, were selected for further analysis (Figure 1A). At the vegetative growth stage, a marked phenotype could be observed after 14-d growth. Both the seedling height and leaf angles were decreased compared with the wild type (Figure 1B). At the reproductive stage, the mutants exhibited a much more compact structure, with reduced plant height and erect leaves (Figures 1C–F). The decreases of mutant leaf angles were more obvious because at this stage the lamina joints of the wild-type plants greatly bended downward whereas the mutants had basically no bending (Figures 1C–F). Surprisingly, all the mutants had increased grain width, with slight change of grain length (Figures 1G–I), which are reminiscent of BR signaling mutant such as *dlt*, suggesting the involvement of SERK2 in BR responses. OsBAK1 is the closest homolog of SERK2 and has been functionally characterized previously (Li et al., 2009; Yuan et al., 2017). We also edited *OsBAK1* gene using a same strategy, and found *osbak1* had a consistent BR-defective phenotypes as reported, including dwarfism, erect leaves, and decreased grain size (Li et al., 2009; Zuo et al., 2014; Yuan et al., 2017). Thus, it appears that SERK2 plays a relatively specific role in BR responses compared to OsBAK1. Given the beneficial effects in the mutants, *SERK2* could be valuable for engineering plant architecture.

### The *serk2* Mutants Have Decreased BR Sensitivity

**FIGURE 2:**
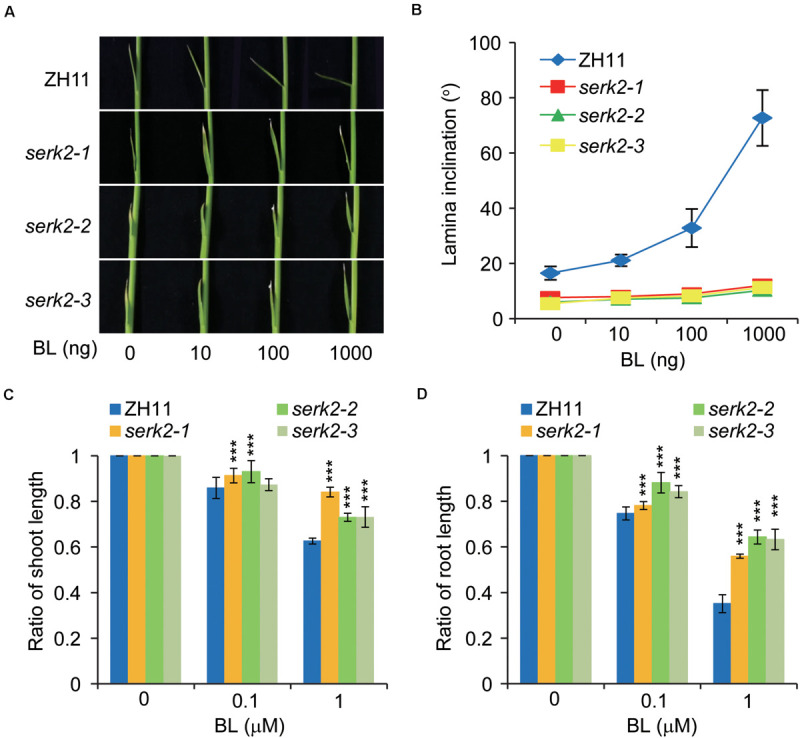
The *serk2* mutants have decreased BR sensitivity. **(A,B)** Lamina bending analysis of the BR sensitivity with statistical data shown in **(B)**. Bars = SD, *n* = 7. **(C,D)** Growth inhibition of the shoot **(C)** and root **(D)** in response to BL in ZH11 and the mutants. Ratios between BL-treated plants and those without BL treatment are shown. Bars = SD, *n* = 15, ***p < 0.001 by *t*-test.

In rice, a high level of active BRs is able to induce leaf bending but inhibit shoot and root growth (Tong et al., 2014). To test the BR sensitivities of *serk2* mutants, we treated plant seedlings with BL and then compared the growth of the three tissues with those of wild type. In lamina bending assay, while the wild type showed gradually enlarged leaf angles along with the increase of BL amount, the *serk2* mutants had basically no response to BL (Figures 2A,B). Similar results were obtained in growth inhibition analysis. The growths of both the shoot and root in the mutants were much less inhibited by BL compared to the cases in the wild type (Figures 2C,D). All these results strongly suggested the critical role of SERK2 in BR responses.

### SERK2 Localizes on Plasma Membrane and Interacts With OsBRI1

**FIGURE 3:**
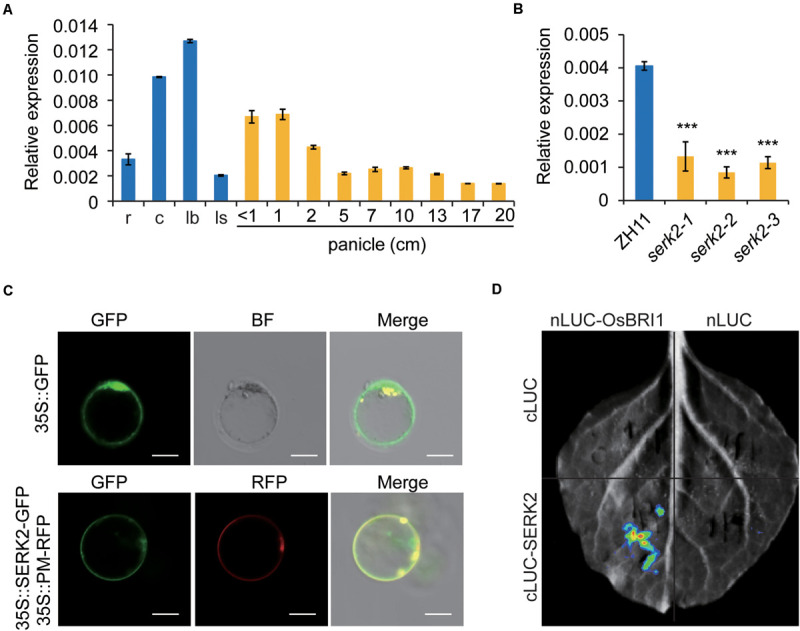
Molecular characterization of SERK2. **(A)** Expression pattern of *SERK2* in various tissues. r, root; c, culm; lb, leaf blade; ls, leaf sheath. Bars = SD, *n* = 3. **(B)** Expression levels of *SERK2* in ZH11 and the mutants. Bars = SD, *n* = 3, ***p < 0.001 by *t*-test. **(C)** Subcellular localization of SERK2, showing a co-localization with plasma membrane marker protein (PM). Empty GFP vector was used as control. Scale bar = 10 mm. **(D)** Split-luciferase complementation analysis showing the interaction between SERK2 and OsBRI1.

Expression analysis by qRT-PCR showed that SERK2 was constitutively expressed in various tissues, with a preference in leaf blade and culm, but less in other tissues (Figure 3A). This expression pattern was somewhat consistent with the mutant phenotypes with main defections in leaf and culm. In addition, *SERK2* had greatly decreased expressions in the mutant (Figure 3B), implying that the dysfunctional *SERK2* transcripts were somehow suppressed in the mutants. However, we failed to detect increased expressions of the two BR synthetic genes, *D2* and *D11*, suggesting that the feedback regulation between BR signaling and synthesis somehow was not significant or not strong enough for detection in *serk2*. We also analyzed the subcellular localization of SERK2 by evaluating the fluorescence of SERK2-GFP fusion proteins in rice protoplast. The results showed that SERK2 was specifically localized on plasma membrane, where can be co-localized with OsBRI1 which serves as a plasma membrane marker (Figure 3C). Furthermore, we tested the potential interaction between SERK2 and OsBRI1 using split-luciferase complementation analysis, and detected the marked signal in tobacco leaves (Figure 3D). Together with the mutant phenotypes, these analyses demonstrated that SERK2 plays a critical role in BR signaling in rice, possibly as a co-receptor of OsBRI1.

### The *serk2* Mutants Show Decreased Survivability Under Salinity Stress

**FIGURE 4:**
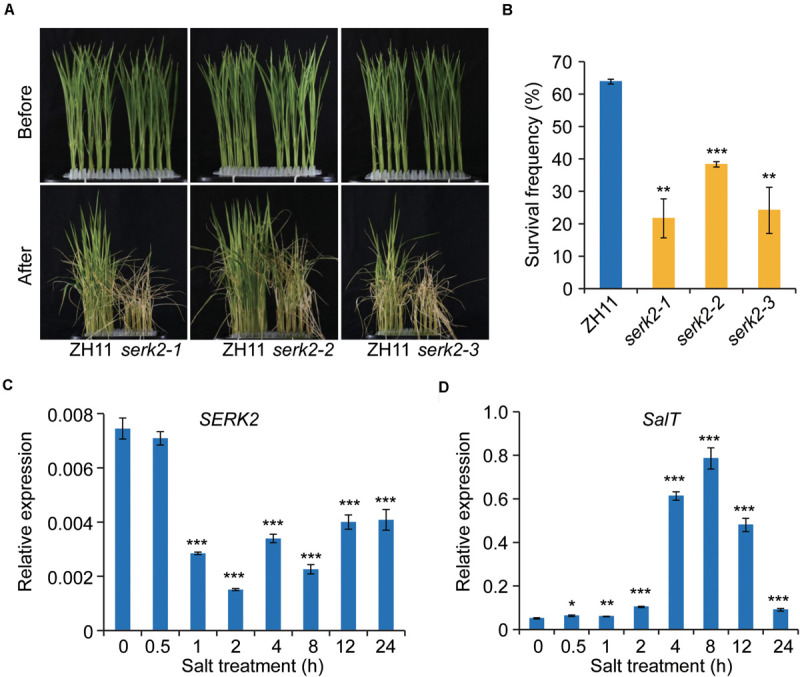
*SERK2* regulates salt stress tolerance and is regulated by salt. **(A)** The *serk2* mutants have decreased surviving frequency after salt treatment. **(B)** Statistical data of the surviving frequency. Bars indicate standard error of the mean values, *n* = 3, ***p < 0.001 and **p < 0.01 by *t*-test. **(C,D)** *SERK2* expression is suppressed by salt treatment. *SalT*, a known salt-induced gene, was used as control. Bars = SD, *n* = 3, ***p < 0.001, **p < 0.01, and *p < 0.05 by *t*-test.

Next, we tested whether the mutant had altered resistance to salt stress. One-week-old seedlings were used for the analysis as at the time the mutant had little change of plant height. Strikingly, after high salt treatment (200 mM NaCl, 6 days) followed with recovery, all the three mutants showed greatly decreased survival frequencies compared to the wild type (Figure 4A). On average, the survival frequency of ZH11 was ∼65%, whereas of the mutant were ∼20–40%, strongly suggesting that SERK2 is required for plant resistance to salt stress (Figure 4B). To test whether *SERK2* is responsive to salt at molecular level, we treated ZH11 with salt, and then analyzed *SERK2* expression at different time points. Strikingly, *SERK2* was greatly suppressed by salt treatment after 1-h treatment, and the inhibitory effect could be kept to 24 h (Figure 4C). As control, expression of *SalT*, a salt induced gene (Zhang et al., 2000), was promoted by salt treatment (Figure 4D). Thus, *SERK2* is involved in salt stress response and positively regulates salt resistance.

### The *serk2* Mutants Show Increased Sensitivity to ABA

**FIGURE 5:**
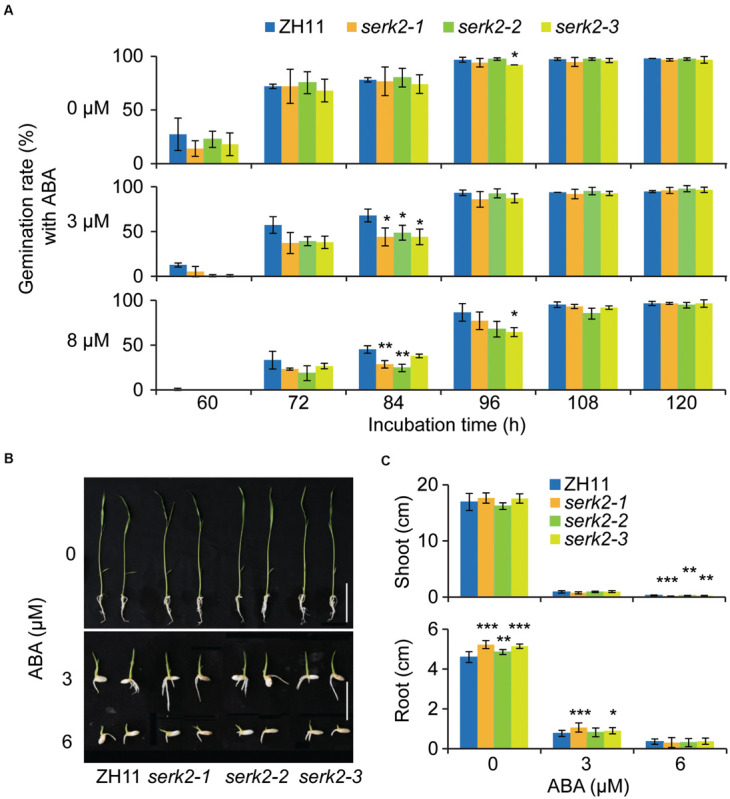
The *serk2* mutants have slightly increased ABA sensitivity. **(A)** Germination test of ZH11 and the *serk2* mutants in response to ABA. Bars = SD, *n* = 3, **p < 0.01, and *p < 0.05 by *t*-test. **(B,C)** Shoot and root growth of ZH11 and the *serk2* mutants in response to ABA. Scale bar = 2 cm in **(B)**. In **(C)**, bars = SD, *n* = 12, ***p < 0.001, **p < 0.01, and *p < 0.05 by *t*-test.

The hypersensitivity of *serk2* mutants to salt prompted us to test whether the plants also have altered sensitivity to ABA, the well-known stress hormone. Without ABA, no significant difference was detected between the mutant and the wild type regarding the seed germination process (Figure 5A). However, when ABA (3 μM or 8μM) was supplemented, all the three allelic mutants showed decreased germination with detectable significance at the certain time points (84 h or 96 h) (Figure 5A), suggesting that the mutant has enhanced ABA sensitivities. Similar results were obtained when evaluating the ABA inhibitory effects on shoot and root growth. The inhibitory effects of ABA (3 μM or 6 μM) on either shoot and root in the mutant were greater than those in the wild type (Figures 5B,C). Given that enhanced ABA response should facilitate plant in cope with stress, these results indicated that the increased salt sensitivity of *serk2* mutants should be independent of ABA, and SERK2 as a BR signaling component could somehow affect ABA responses.

### The *serk2* Mutants Are Hypersensitive to Salt Stress at Molecular Level

**FIGURE 6:**
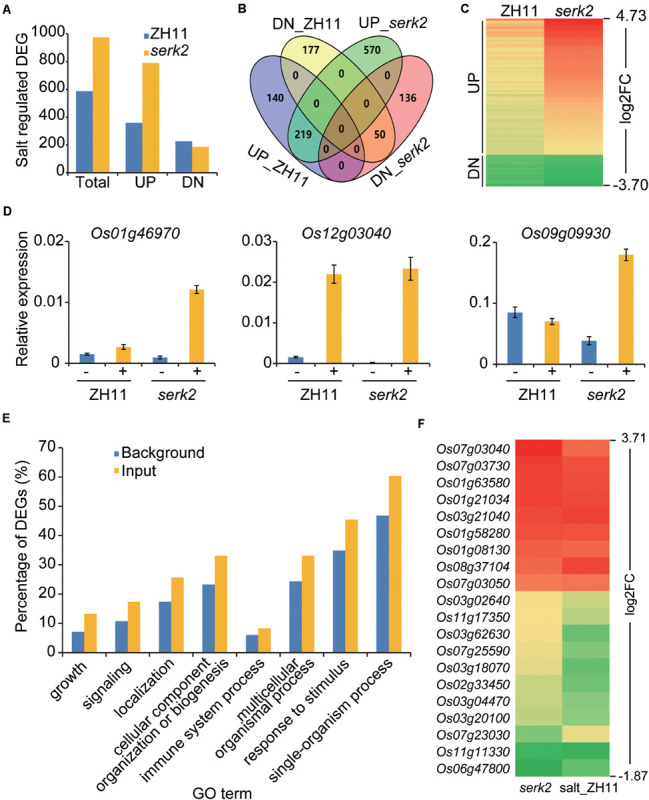
The *serk2* mutants are more sensitive to salt at molecular level. **(A)** Salt-regulated DEGs in ZH11 and *serk2* identified by transcriptome analysis respectively. UP, upreuglated DEGs; DN, downregulated DEGs. **(B)** Overlapping analysis of the salt-regulated DEGs in ZH11 and *serk2*. DEG numbers are indicated in each section. This Venn diagram was generated online (https://bioinfogp.cnb.csic.es/tools/venny/). **(C)** Heatmap of the overlapping DEGs showing the logarithmic values of the fold changes (log2FC) in ZH11 and *serk2* upon salt treatment, illustrating the FC in *serk2* is generally higher than in ZH11. **(D)** Quantitative RT-PCR analysis of the three selected DEGs ± indicate with/without salt. Bars = SD, *n* = 3. **(E)** GO enrichment analysis of DEGs identified from *serk2* (input). Top eight GO terms identified in biological processes were shown. The percentages of the gene numbers in each GO term accounting for all gene numbers were shown as background. **(F)** Heatmap of the overlapping DEGs identified in both *serk2* and salt-treated ZH11 (salt_ZH11). Log2FCs were used for generating the map, illustrating the consistent expression tendencies of all the DEGs in the two samples.

To verify the roles of SERK2 in stress responses, we performed transcriptome analysis to identify the salt-regulated genes in the wild type and the mutant respectively. Four samples, including ZH11, *serk2-1*, ZH11 treated with salt (200 mM NaCl, 8 h), and *serk2-1* treated with salt, were prepared and the seedling shoots were collected as materials for the analysis. The DEGs in salt-treated samples, namely salt-regulated genes, were used for the comparison (Supplementary Tables 2, 3). As results, we identified 586 salt-regulated genes in ZH11 background, but identified 975 in *serk2* background (Figure 6A). Intriguingly, most of these DEGs were up-regulated ones, and consistently, salt treatment apparently had upregulated more genes in *serk2* than in ZH11 (Figure 6A). We identified 329 overlapping DEGs between salt regulated genes in ZH11 and *serk2*. Notably, all of them were consistently regulated (Figure 6B). Among the salt upregulated DEGs in ZH11, 77.7% (279/359) were also upregulated by salt in *serk2* (Figure 6B). Importantly, the change folds of most overlapping DEGs in *serk2* were higher than those in ZH11 (Figure 6C). We selected three genes for verification, and found these genes indeed were much more obviously induced in the mutant than in the wild type (Figure 6D). Taken together, these analyses further confirmed that loss-of-function of *SERK2* enhances susceptibility of the mutant to salt treatment at molecular level.

### Altered Expression of Salt-regulated Genes in the *serk2* Mutants

Compared to ZH11, we identified 129 DEGs from *serk2*, including 72 upregulated and 57 downregulated. As revealed by gene ontology (GO) analysis, these DEGs were markedly enriched in biological processes such as “growth” and “signaling” (Figure 6E), consistent with the roles of SERK2 in regulating plant growth and development and hormone signaling. In addition, the enrichment in GO terms such as “immune system process” and “response to stimulus” indicated that SERK2 is also involved in plant immunity, as has been reported previously (Chen et al., 2014), and stress responses, as revealed in this study (Figure 6E). Moreover, 20 of the DEGs were also differentially regulated in the salt treated ZH11 samples (salt_ZH11, Figure 6F). Notably, all these 20 DEGs, had consistent expression tendency, either upregulated or downregulated, in *serk2* and salt_ZH11 (Figure 6F). Thus, it appeared that loss-of-function of *SERK2* has more or less activated salt responses even without salt treatment. One possibility is that these genes represent a subset of salt responsive genes that are unfavorable for plant adaptability to salt stress.

### Overexpression of *SERK2* Enhanced Grain Size and Salt Resistance With Little Effect on Plant Architecture

**FIGURE 7:**
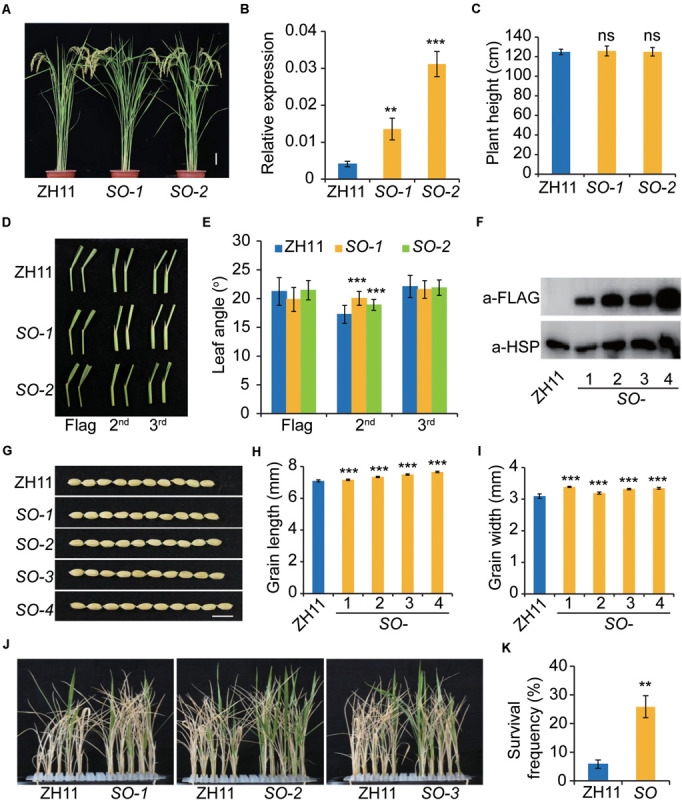
Overexpression of *SERK2* simultaneously enhances grain size and salt resistance without affecting plant architecture. **(A)** Plant architecture of the *SERK2*-overexpressing plants. Scale bar = 10 cm. **(B)** Expression levels of *SERK2* in ZH11 and the transgenic plants. Bars = SD, *n* = 3, ***p < 0.001 and **p < 0.01 by *t*-test. **(C)** Statistical data of the plant height. Bars = SD, *n* = 15. ns, no significant difference. **(D)** Leaf angles in ZH11 and the transgenic plants. Top three leaves are shown. *SO, SERK2-*overexpression lines. **(E)** Statistical data of the leaf angles. Bars = SD, *n* = 20, ***p < 0.001 by t-test. **(F)** SERK2-FLAG fusion proteins are accumulated in the transgenic plants. Detection of HSP is used as reference. **(G)** Grain morphology of ZH11 and the transgenic plants. Scale bar = 1 cm. **(H,I)** Statistical data of grain length **(H)** and grain width **(I)**. Bars = SD, *n* = 50, ***p < 0.001 by *t*-test. **(J,K)** Salt treatment showing the three independent transgenic lines have increased survival frequencies after salt treatment. Bars = SD, *n* = 3, **p < 0.01 by *t*-test.

To gain more insight into *SERK2* function, we introduced a construct expressing SERK2-Flag fusion protein into ZH11 and obtained a number of transgenic lines. However, we failed to observe a clear morphological difference compared to the wild type (Figure 7A). In two lines with enhanced *SERK2* expression, no difference was detected in term of plant height (Figures 7B,C). Regarding the leaf angle, we can only detect a slight increase of the second leaf (the flag leaf was counted as the first leaf), but not in the first and the third ones (Figures 7D,E). Immunoblotting analysis further confirmed the accumulation of SERK2-FLAG fusion proteins in both the two lines as well as two additional lines (Figure 7F). Notably, all these transgenic lines evaluated showed obviously increased grain length and width (Figures 7G–I). We also tested the stress adaptability of three lines and found that all of them showed consistently increased survival frequencies after high salt treatment (200 mM NaCl, 8 d) (Figures 7J,K). Thus, our results suggested that overexpression of *SERK2* is able to simultaneously promote grain size and stress resistance. The little effect on the plant architecture further enhances the feasibility of utilization of the gene for crop improvement.

### SERK2 Protein Is Greatly Induced by Salt Stress

**FIGURE 8:**
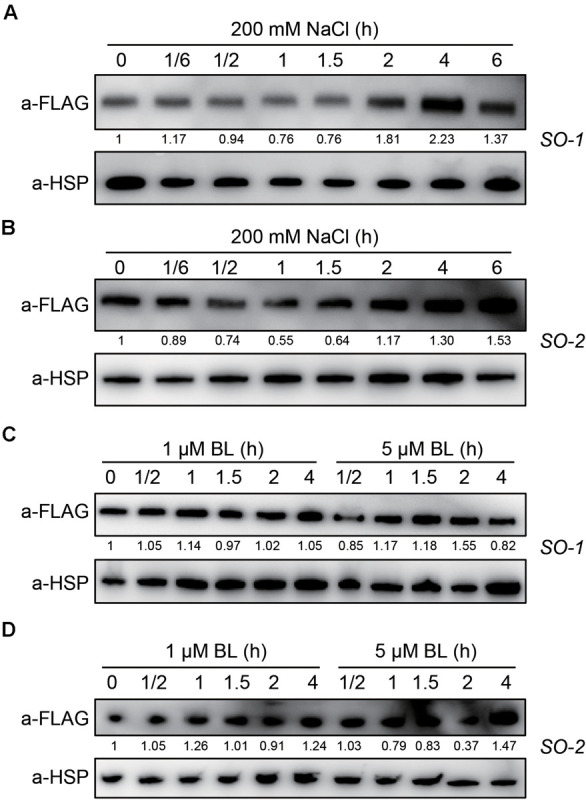
SERK2 protein is greatly induced by salt treatment. **(A,B)** Evaluation of salt stress on SERK2-FLAG protein levels using two independent transgenic lines. Detection of HSP is used as reference. **(C,D)** Evaluation of BL on SERK2-FLAG protein levels using two independent transgenic lines. Detection of HSP is used as reference. Numbers indicate the relative intensities of each band, representing the relative abundances of SERK2-FLAG proteins.

Since SERK2 positively regulates salt tolerance but is suppressed by salt at transcription level, we are curious how SERK2 is regulated by salt at protein level. When the overexpression lines were treated with high salt for different times, the SERK2-FLAG fusion proteins showed a dynamic expression pattern. Upon treatment for 1 h, the protein abundance was slightly suppressed by salt (Figures 8A,B). After that, the protein was gradually increased and was accumulated to a high level after 4- or 6-h-treatment (Figures 8A,B). However, when treated with BL, the fusion proteins showed unaltered or only slightly altered levels (Figures 8C,D). For both treatments, we obtained consistent results using two independent lines for the analysis. Thus, it appears that SERK2 is slightly inhibited and then greatly induced upon stress treatment. This result further demonstrates the involvement of SERK2 in salt stress responses. The eventually highly accumulation of the protein could favor the plant in cope with the adverse condition.

## Discussion

BRs regulate many important agronomic traits such as plant height, leaf angle and grain size. However, the regulation of these traits is not simultaneously beneficial for crop improvement in the view of breeders. For example, deficiency of BRs could decrease plant height and leaf angle, which are associated with increased lodging resistance and photosynthetic efficiency, allowing high density planting, a major cultivation strategy for obtaining high yield (Sakamoto et al., 2006). However, BR deficiency could also impair grain size or weight, leading to reduced grain yield. Similarly, BR overdose could increase grain weight but this is usually accompanied with a loose plant architecture that is not preferred by farmers (Wu et al., 2008). To deal with this problem, one feasible approach is to identify specific BR components. Manipulation of this kind of BR components could obtain the desired traits without or with little effect on other traits (Tong and Chu, 2018). Considering each BR signaling step is executed by a family of genes, the genes within the family could have differential function or expression pattern. In this case, it’s worthy to screen these family genes to identify the specific ones that regulate certain traits. Here, we showed that compared to OsBAK1, its homolog SERK2 functions more specifically in regulating leaf bending. Knockout of *OsBAK1* led to small grains (Yuan et al., 2017), but knockout of *SERK2* resulted in even increased grain size. Although both mutants show erect leaves, apparently *serk2* is a more applicable candidate for crop improvement. This result is also consistent with the gene expression pattern. According to the public database^2^, *OsBAK1* has the highest expression in panicles, whereas *SERK2* has the highest expression in leaves.

Since BRs also play a critical role in stress responses, stress is another important aspect that should be considered when manipulation of BR genes. Our evaluation suggests that defective of BR signaling could decrease plant resistance to salt stress. Unlike most of the previous studies focusing on the downstream of BR signaling pathway (Cai et al., 2014; Hu and Yu, 2014; Wang et al., 2018; Ye et al., 2019), our study revealed that early BR signaling on plasma membrane is involved in stress responses. High salt is able to greatly induce SERK2 protein accumulation, suggesting that the adverse condition induces SERK2 accumulation to enhance early BR signaling on plasma membrane in favor of the anti-stress response. In addition, it appears that the slightly increased ABA sensitivity could not explain the hypersensitivity of *serk2* to salt stress, implying the existence of an ABA-independent role of BR signaling in the stress responses. On the other hand, we show that overexpression of *SERK2* increases grain size as well as salt stress resistant ability. Importantly, the transgenic lines have little alteration of plant architecture including plant height and leaf angle, providing an alternative approach for *SERK2* utilization.

High yield and stress resistance are usually conflicting with each other due to the internal unknown compensation mechanism. However, high yield and high resistant crops are long-term breeding goals and also prime breeding targets. Phytohormones usually play important roles in both plant growth and development and stress responses. The dual roles of SERK2 in grain size and salt stress indicate that BRs have great potential for simultaneously improvement of grain yield and stress resistance, at least to salinity stress. Intriguingly, transcriptome analysis revealed that a subset of salt-regulated genes have consistently altered expression in *serk2* mutant, indicating that SERK2 regulates these genes to enhance plant resilience. Since SERK2 promotes BR signaling at the same time, one possibility is that these genes are also BR responsive genes by which BRs enhance salt resistance.

Considering the increased grain size in *SERK2*-overexpressing plants, the increased grain width in *serk2* mutants could be due to a compensation effect among the family members or feedback regulation on BR hemostasis in plants. Similarly, loss-of-function of *DLT*, a positive BR signaling regulator, also led to increased width, whereas overexpression of *DLT* resulted in increased grain size (Tong et al., 2009, 2012). These analyses, together with the previous studies (Tong et al., 2014; Xiao et al., 2017), suggested the existence of a highly elaborated BR balance system in plant, which may provide a potential explanation for the unaltered plant architecture in *SERK2*-overexpressing plants. Elucidation of this balancing system is critical for uncoupling different BR functions, thus facilitates the utilization of BR for crop improvement.

## Conclusion

Salt stress induces SERK2 accumulation to enhance early BR signaling on plasma membrane in favor of the anti-stress response. Knockout of *SERK2* led to compact structure accompanied with decreased salt tolerance, whereas overexpression of *SERK2* is able to simultaneously enhance grain size and salt resistance without affecting plant architecture.

## Data Availability Statement

The datasets presented in this study can be found in online repositories. The names of the repository/repositories and accession number(s) can be found below: National Genomics Data Center (https://bigd.big.ac.cn/) (accession: CRA003500).

## Author Contributions

ND performed most experiments with the assistance of the others. WY and DL assisted in the protein analysis. XZ and ZY assisted in phenotype analysis. WH and JL provided assistance in expression and transgenic analyses. YY participated in the transcriptome analysis. WM and MN assisted in stress treatment. ND and HT analyzed the data and wrote the manuscript. HT conceived and supervised the study. All authors contributed to the article and approved the submitted version.

## Conflict of Interest

The authors declare that the research was conducted in the absence of any commercial or financial relationships that could be construed as a potential conflict of interest.

## Footnotes

1 http://bioinfogp.cnb.csic.es/tools/venny/

2 https://ricexpro.dna.affrc.go.jp/
